# Impact of Farming System on Soil Microbial Communities Associated with Common Bean in a Region of Northern Spain

**DOI:** 10.3390/plants14091359

**Published:** 2025-04-30

**Authors:** Marta Suarez-Fernandez, Juan Jose Ferreira, Ana Campa

**Affiliations:** Plant Genetic Group, Regional Service for Agrofood Research and Development (SERIDA), 33300 Villaviciosa, Spain; marta.suarezfernandez2@asturias.org (M.S.-F.); juanjose.ferreirafernandez@asturias.org (J.J.F.)

**Keywords:** common bean, metabarcoding, organic farming, conventional farming, microbial diversity, microbial networks

## Abstract

Agricultural soil microbiomes play a crucial role in the modification and maintenance of soil properties such as soil fertility, nutrient availability, and organic matter decomposition. This study assessed the influence of organic and conventional farming practices on soil microbiomes associated with common bean (*Phaseolus vulgaris* L.) at the field scale in Northern Spain. Metabarcoding techniques were used to compare both microbial communities. Alpha and beta diversity analyses revealed that organic soils supported richer fungal communities with a higher species evenness, whereas conventional soils were abundant in prokaryotes. Taxonomic assignment of the observed Operational Taxonomic Units (OTUs) identified a total of 1141 prokaryotic and 622 fungal taxa. Among these, 200 prokaryotic and 113 fungal OTUs showed significant differences in response to different farming practices. This classification allowed the establishment of a core microbial community associated with the common bean crop, comprising 594 prokaryotic OTUs classified into 11 phyla, and 256 fungal OTUs classified into 11 phyla. Functional analyses indicated that organic farming promoted a broader range of prokaryotic functions related to nitrogen metabolism, stronger positive interactions between fungi and bacteria, a higher abundance of beneficial microorganisms, such as biocontrol fungi and mycorrhizae, and greater overall microbial stability. In contrast, conventional soil showed a higher prevalence of potentially phytopathogenic fungi and more complex, competitive microbial interactions. These results highlight the effect of the farming system on the diversity and microbial composition of the soils associated with bean crops in Northern Spain. While further research in different climatic regions and crop systems is essential, these findings underscore the potential of organic farming to improve soil diversity and enhance microbial network interactions.

## 1. Introduction

Modern agricultural practices pursue sustainable food production. Soil is a fundamental component of food production, playing crucial roles in nutrient cycling, water management, pest and disease control, and supporting biodiversity. In addition, soils contribute to climate change mitigation because of their role in carbon sequestration [1,2]. Soils serve as complex habitats for many organisms, including bacteria, fungi, protists, plants, and invertebrates, which collectively contribute to soil health and ecosystem functioning [3]. Microorganisms interact with each other and the soil environment in various ways. These interactions can be symbiotic, competitive, or antagonistic and play a key role in important soil functions such as nutrient cycling, soil structure formation, plant growth promotion, and the breakdown of organic matter. These interactions are referred to as soil microbial networks. The complexity of these networks increases with microbial richness and contributes to enhanced ecosystem sustainability. In contrast, simpler or less diverse networks, often linked to lower microbiome richness, are associated with reduced contributions to ecosystem functions [4,5]. In this regard, soil health has been defined as “the ability of the soil to sustain the productivity, diversity, and environmental services of terrestrial ecosystems” [6]. Healthy soils are characterized by diverse and active microbiomes that play a vital role in nutrient cycling, disease suppression, and maintenance of the soil structure [7]. The resilience of an ecosystem—its ability to recover from disturbances and adapt to changing conditions—relies heavily on the diversity and functionality of its microbial communities.

Soil microorganisms (syn. soil microbiome) play distinct functional roles that reflect how they interact with each other. Based on their habitat, they can be classified as generalists, which inhabit a wide variety of environments, or specialists, which are adapted to specific habitats and are often restricted to them [8]. Regarding nutritional mode, prokaryotes can be grouped functionally: phagotrophs consume organic particles and help decompose dead plant and animal matter; phototrophs, such as photosynthetic bacteria, use light energy to produce food and support nutrient balance in the soil; parasites and plant pathogens, such as *Xanthomonas* species, extract nutrients by infecting and often damaging plants; saprotrophs, such as *Bacillus* species, decompose dead organic matter and play a crucial role in nutrient cycling; and mixotrophs use both organic and inorganic compounds, making them highly versatile in nutrient acquisition [9,10]. Similarly, fungi may be classified into three main trophic modes [11]: pathotrophs, such as *Rhizoctonia* or *Phytophthora* species that derive nutrients by damaging plant cells, often leading to root rot and plant disease; symbiotrophs, like mycorrhizal fungi, which form mutualistic associations with roots, exchanging nutrients and enhancing plant growth; and saprotrophs, such as *Trichoderma* or *Aspergillus*, that break down organic matter aiding in decomposition and nutrient recycling. The distribution of these functional groups, along with the networks established among microorganisms, can introduce new functions that they do not have separately through synergistic interactions, playing a crucial role in nutrient cycling for crop cultivation [12,13,14].

Metabarcoding is a robust method for assessing the taxonomic composition and diversity of soil microbiomes and provides insights into the variety of microbial species present in soil, water, or food [15]. Metabarcoding allows for the simultaneous identification of multiple taxa within a single sample by amplifying DNA, sequencing specific amplicons (barcodes), and aligning sequences with reference databases [16]. One of the key advantages of metabarcoding is its ability to analyze large and complex microbial communities in a cost-effective and efficient manner. Additionally, it allows for the detection of microorganisms that are difficult or impossible to culture in a laboratory. This technology has been successfully applied to investigate microbiome diversity across soils, detecting over 79,000 bacterial and 25,000 fungal Operational Taxonomic Units (OTUs) in 715 sites across 24 European countries [17].

Legumes play a crucial role in maintaining and improving soil health, as well as restoring degraded soils. This is primarily due to their symbiotic relationship with nitrogen-fixing bacteria, which enhances soil fertility and forms complex networks of biological activity [18]. Legumes are valued not only for their immediate high yields and nutritional value but also for their ability to enrich soils for subsequent crops. Common bean (*Phaseolus vulgaris* L.) is one of the world’s most widely consumed grain legumes, with seeds that are a significant protein source [19], containing phenolic compounds that act as antioxidants and provide health benefits [20].

There is growing evidence that different farming systems, such as conventional and organic, affect soil microbial communities [21]. However, our understanding of the response of microbial diversity to these practices remains limited. Conventional and traditional farming rely heavily on the regular application of synthetic fertilizers, pesticides, and herbicides to maximize crop yields. Although this approach can be effective in the short term, it is expected to disrupt the soil microbes and reduce biodiversity. In contrast, organic agriculture emphasizes the use of natural inputs and avoids synthetic chemicals [22]. Therefore, we hypothesized that organic practices may support a balanced and diverse soil microbiome, which may contribute to the formation of diverse and interconnected soil microbial networks.

This study aimed to evaluate the putative impact of organic and conventional farming practices on the prokaryotic and fungal diversity of soils associated with common bean crops located in Northern Spain and to determine whether changes in microbial diversity influence the structure and complexity of soil microbial networks.

## 2. Results

### 2.1. Soil Physicochemical Features and Environmental Data

Thirty-eight physicochemical characteristics were evaluated in 2022 and 2023 (Table S1) for the soil managed with conventional practices (named conventional soil, CS) and for the soil managed with organic practices (named organic soil, OS). Both soils had similar pH values, although conductivity was higher in CS. OS showed a higher abundance of nitrate and nitric nitrogen (circa 60% higher), total nitrogen (ca. 30% higher), potassium (ca. 70% higher), assimilable potassium (ca. 50% higher), and assimilable manganese (ca. 100% higher) than CS. In contrast, CS showed a higher content of sulfate (ca. 90% higher), sodium (ca. 110% higher), calcium (ca. 70% higher), magnesium (ca. 170% higher), assimilable phosphorus (ca. 80% higher), assimilable zinc (ca. 50% higher), and sodium adsorption ratio (ca. 60% higher).

Soil environmental data were collected for both soils in 2022 and 2023 (Figure S1). The soil temperature was slightly higher in CS. Moisture, on the other hand, remained stable in CS in 2023, while in OS, it decreased between April and September. In addition, CS showed a greater incidence of days with high temperatures (above 25 °C) than OS, mainly from June to September.

### 2.2. Prokaryotic (16S) and Fungal (ITS) Sequencing

In both years, contaminant amplicons were either absent in most samples or at least one order of magnitude less abundant than those in the negative control. Table 1 summarizes the 16S rRNA and ITS sequencing data. The initial mean number of input reads ranged from 86,807 to 120,179 for 16S sequencing and from 41,308 to 160,772 for ITS sequencing. After filtering, denoising, and merging, the average OTUs ranged from 352 to 634 for 16S and from 147 to 260 for ITS. Two biological replicates from 2023 (CS4 and OS5) were excluded from the 16S analysis because of anomalous OTU values.

The fungi/prokaryote ratio in CS was 0.26 in 2022 and 0.32 in 2023. This ratio was approximately doubled in OS, increasing to 0.53 and 0.62 in 2022 and 2023, respectively. These results indicate that while prokaryotes were more abundant in CS, OS supported a more balanced community structure between fungi and prokaryotes, with a higher relative abundance of fungi.

### 2.3. Alpha and Beta Diversity

Rarefaction plots (Figure S2) showed that the sequencing depth and subsampling size achieved in both years were sufficient to capture the complete diversity present in the 16S and ITS communities, reaching a plateau for the observed OTUs.

**Figure 1:**
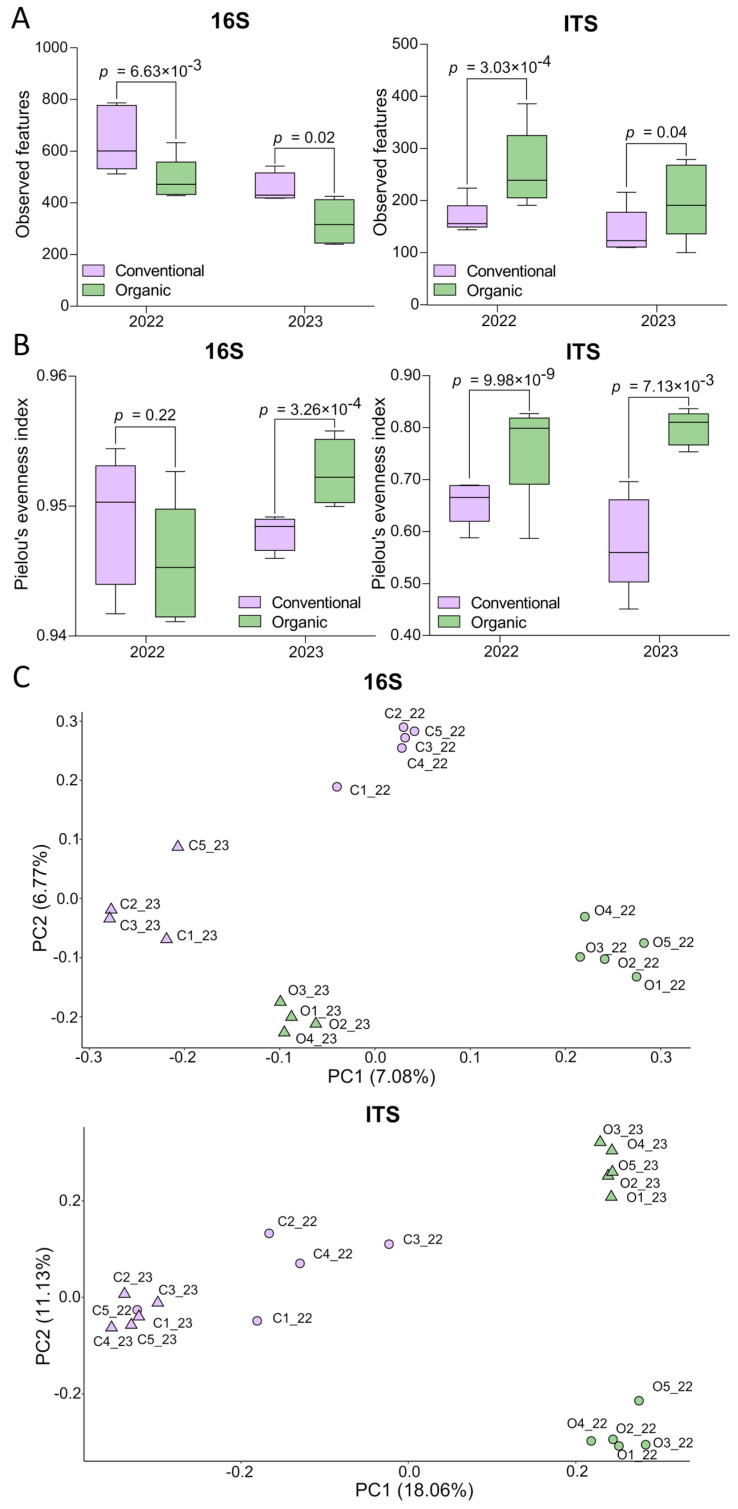
Variation in the composition of prokaryotic (16S) and fungal (ITS) communities in the conventional soil (CS) and the organic soil (OS) per year. (**A**) Alpha diversity measured as richness: total observations of sequenced samples. (**B**) Alpha diversity measured as evenness: Pielou’s index. (**A**,**B**) The boxplots include four to five independent biological replicates and p values according to the t-test and are indicated for all comparisons. (**C**) Beta diversity: PCoA from Jaccard’s distances. Dots: Year 2022. Triangles: Year 2023. The distances between the triangles and the dots represent the ecological distances between the samples.

Alpha diversity was measured using three parameters: richness, evenness, and the Shannon index. Regarding richness, the 16S observations were higher in CS than in OS, whereas the ITS observations showed the opposite trend (Figure 1A). The statistical analysis of richness in response to farming system and year is shown in Table 2. For 16S, significant differences in the number of observed OTUs were detected for both farming system and year. For ITS, significant differences were observed only for farming system but not for year. The interaction between farming and year was not significant for either the 16S or ITS sequences. Evenness, measured as Pielou’s index, is represented in Figure 1B. For 16S, significantly higher values were observed for OS in 2023, but no significant differences were observed in 2022. For ITS, significant differences were observed in both years, with higher values for OS than for CS. The statistical analysis of evenness in response to the farming system and year is shown in Table 2. For 16S, only the interaction between farming and year showed a significant impact on evenness, while for ITS, only farming practices had a significant effect.

The Shannon diversity index analysis in bacterial communities ranged between 2.74 and 4.65 and revealed no significant differences (Table 2). Neither year nor interaction between farming and year showed significant effects on bacterial diversity. The lack of significant differences was consistent throughout the study period. In contrast, the Shannon diversity index in fungal communities ranged between 5.28 and 6.34 and exhibited significant differences according to farming (Table 2). Additionally, neither year nor interaction between treatment and year had a significant effect on fungal diversity.

Beta diversity, which measures the variation in community composition between samples, was estimated using Jaccard’s similarity coefficients and visualized using PCoA (Figure 1C). The scatter plots show high similarity between the samples according to the farming system and year. PCoA revealed two principal components from the 16S data, explaining 7.08% and 6.77% of the variation. For the ITS data, PCoA revealed two principal components explaining 18.06% and 11.13% of the variation. Statistical analysis of beta diversity revealed significant differences in farming, year, and interaction farming year in 16S and ITS OTUs (Table 2).

### 2.4. Taxonomy

Taxonomic assignment of the observed OTUs was performed. The observed composition of the mock community included in the 16S analysis was consistent with the expected theoretical composition in both years, validating the library preparation process.

The analysis revealed 1141 bacterial and archaeal species, with 958 in 2022 and 777 in 2023 (Table S2). Among them, 200 showed significant differences in abundance between CS and OS: 181 in at least one year and 19 in both years (Table 3), many corresponding to uncultured bacteria in the SILVA database. A total of 594 species were consistently observed in both years, accounting for at least 52% of the total prokaryotic diversity, and were considered the core population of prokaryotes associated with the common bean crop (Table S2). For fungi, 622 species were detected, with 455 species detected in 2022 and 423 in 2023 (Table S3). Statistical analyses revealed that 113 species showed significant differences in abundance between CS and OS: 91 in at least one year and 23 in both years (Table 4). A total of 256 species were consistently observed in both years, accounting for at least 41% of the total fungal diversity, and were considered the core population of fungi associated with the common bean crop (Table S3).

### 2.5. Differences in Microbial Communities and Functional Analyses

**Figure 2:**
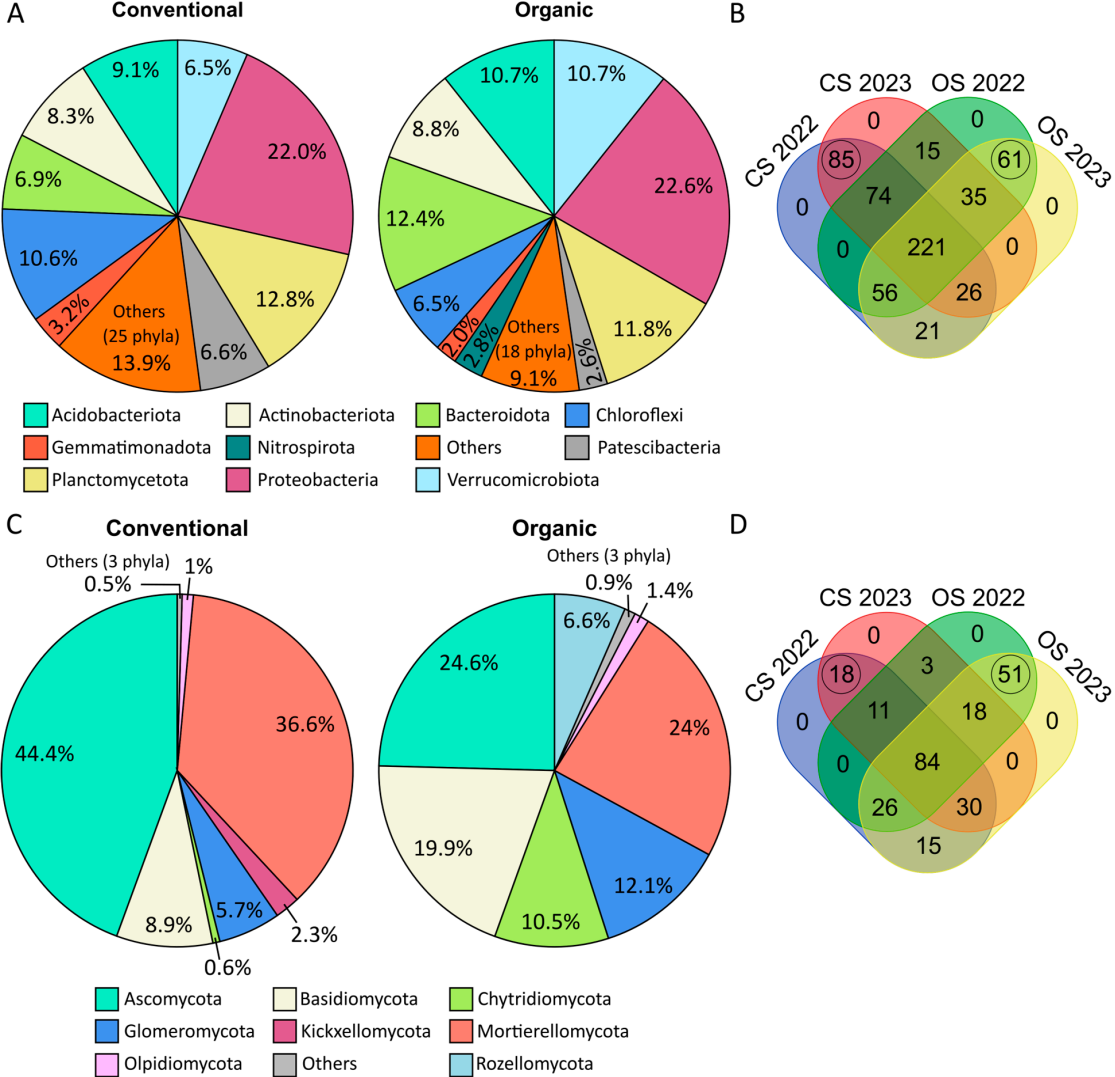
Microbial communities in conventional soil (CS) and organic soil (OS). (**A**) Doughnut plots illustrating bacterial phyla diversity. Phyla with less than 2% abundance are grouped under “Others”. (**B**) Farming distribution of the 594 common prokaryotic species in 2022 and 2023. Species exclusively observed in each soil (85 in CS and 61 in OS) are remarked. (**C**) Doughnut plots illustrating fungal phylum diversity. Phyla with less than 0.5% abundance are grouped under “Others”. (**D**) Farming distribution of the 256 common fungi species in 2022 and 2023. Species exclusively observed in each soil (18 in CS and 51 in OS) are remarked.

**Figure 3:**
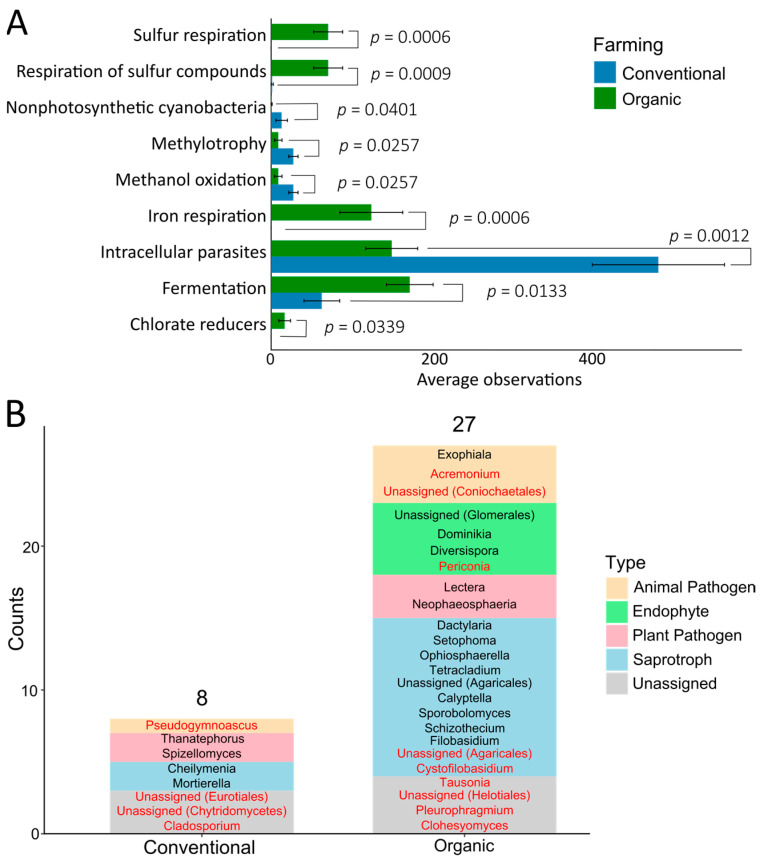
Role of microbial communities in conventional soil (CS) and organic soil (OS). (**A**) Functions annotated in the species exclusively observed in CS or OS that showed significant differences (according to Mann–Whitney U test, *p* < 0.05). The remaining functions are shown in Figure S3. (**B**) Fungal lifestyles observed in the species exclusive to each soil type according to FUNGuild. The guilds assigned with a “possible” confidence ranking are highlighted in red color and should be carefully considered for discussion. For more information, see Table S3.

The 594 prokaryotic species consistently observed in both years (core population) were classified into 34 phyla; 28 were present in both CS and OS, and six were exclusive to CS: Dadabacteria, Entotheonellaeota, FCPU426, Hydrogenedentes, SAR324 clade, and WS2. The relative composition of phyla (Figure 2A) showed that Chloroflexi, Gemmatimonadota, and Patescibacteria, among others, were more abundant in the CS. In contrast, the phyla Acidobacteriota, Bacteroidota, and Verrucomicrobiota were more abundant in OS. Nitrospirota was present only in OS. At the species level, 85 prokaryotic species were exclusive to CS, whereas 61 were exclusive to OS (Figure 2B). General functional analysis with the Functional Annotation of Prokaryotic Taxa (FAPROTAX) revealed that 178 prokaryotic taxa were present in the FAPROTAX database, representing 24.86% of the unique prokaryotic OTUs observed in this study. When classifying observations by the farming system, the functional analyses revealed 44 different functions (41 in conventional farming and 37 in organic farming, Figure S3), and 9 of these functions showed significant differences between CS and OS (Figure 3A).

Regarding fungi, the 256 species consistently observed in both years (core population) were classified into 11 phyla that showed different abundances between CS and OS (Figure 2C). More than 90% of the CS composition corresponded to fungi of three phyla: Mortierellomycota, Ascomycota, and Basidiomycota. The remaining phyla were relegated to lower percentages of contribution to the ecosystem. In contrast, OS showed greater fungal diversity, with 90% of the species being represented in six phyla with similar proportions: Ascomycota, Basidiomycota, Mortierellomycota, Glomeromycota, and Chytridiomycota. The phyla Basidiomycota, Glomeromycota, and Chytridiomycota were considerably more abundant in the OS than in the CS. At the species level, 84 were detected the two years in both, CS and OS, accounting for 33% of 256 common species. Interestingly, 18 species were exclusive to CS and 51 species were exclusive to OS (Figure 2D). OS exhibited a greater variety of trophic lifestyles than CS, highlighting the presence of endophytes, mycorrhizae, and animal or fungal parasites (Figure 3B). Parasites are generally endophytes or saprotrophs that protect plants from pests and diseases caused by insects or pathogenic fungi. In contrast, the CS was mainly enriched with saprotrophic fungi.

### 2.6. Interactions Between the Main Fungal and Prokaryotic Phyla

**Figure 4:**
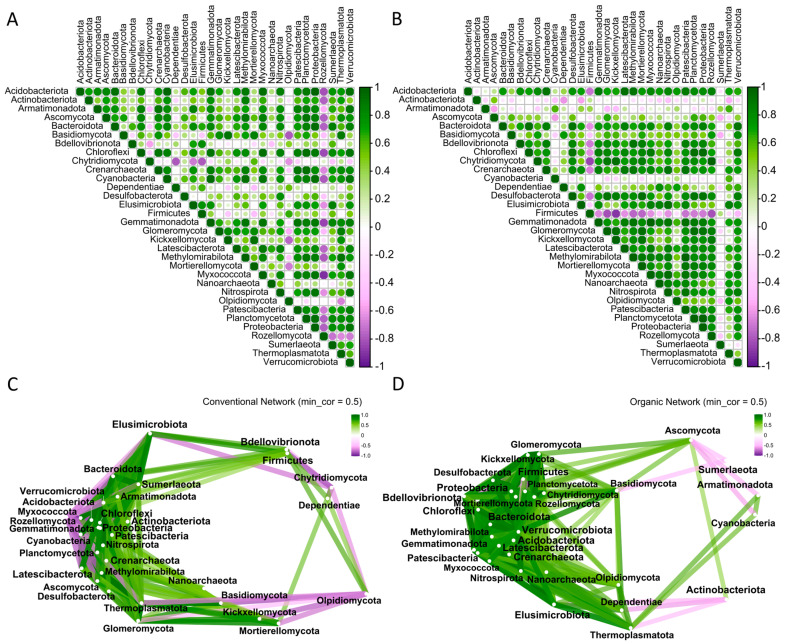
Phyla correlations and networks of the 33 phyla commonly detected in 2022 and 2023. Spearman correlation matrices in (**A**) conventional soil (CS) and (**B**) organic soil (OS). The size of the dot on the graph is directly proportional to the correlation numeric value. For more information on the statistics of the different correlation values, see Table S4. The networks for those 33 phyla in (**C**) CS and (**D**) OS. The intensity of the line is directly proportional to the absolute value of the interaction. Only interactions with a coefficient greater than 0.5 are shown.

Correlation analyses were performed to determine the significant interactions between fungal and bacterial phyla in CS and OS. For this analysis, absolute observations of common phyla detected in 2022 and 2023 were considered, and phyla with residual observations (two or fewer observations across biological replicates) were excluded to facilitate the study. After filtering, 33 phyla were kept for analyses. The Spearman correlation matrix (Table S4) showed distinct interaction patterns in CS (Figure 4A) and OS (Figure 4B). In CS, Rozellomycota exhibited remarkable negative and significant correlations with most other phyla, except for Chytridiomycota, Dependentiae, and Olpidiomycota. Among prokaryotic phyla, several—including Bacteroidota, Chloroflexi, Crenarchaeota, Cyanobacteria, Gemmatimonadata, Methylomirabilota, Nitrospirota, Patescibacteria, Plactomycetota, Proteobacteria, Sumerlaeota, Thermopasmatota, and Verrucomicrobiota—displayed significant positive correlations, highlighting their interdependence in CS.

Firmicutes showed the highest number of significant negative correlations with OS, followed by Actinobacteriota, Sumerlaeota, and Armatimonadota. Most of the remaining fungal and bacterial phyla in OS were positively correlated, reflecting a higher prevalence of cooperative interactions.

The networks were investigated to visualize the differences between the edge relationships. In CS network, both positive and negative interactions were observed (Figure 4C), with several phyla forming complex relationships. Negative interactions could indicate that some phyla are sensitive to conventional practices and could be displaced by more resistant ones. Rozellomycota has emerged as a key phylum, showing a considerable number of connections, with both positive and strong negative interactions with multiple prokaryotic phyla. This suggests that Rozellomycota may play a pivotal role in shaping the microbial communities through competition or exclusion. Chytridiomycota, although less than Rozellomycota, also displayed several interactions, albeit with weaker negative correlations, suggesting a less specialized role in the network. Proteobacteria occupied a central position in the network, interacting with multiple phyla. Actinobacteriota was also highly connected, whereas Firmicutes demonstrated versatility in microbial interactions. In contrast, the OS network was dominated by positive correlations (Figure 4D). Ascomycota showed a central and highly connected position in the network, suggesting its dominance in the OS ecosystem and the promotion of cooperative interactions. Similar to CS, Rozellomycota maintained multiple strong positive correlations, underscoring its importance in the microbial community. Proteobacteria remained a central phylum with robust positive connections, indicating its ecological significance. Planctomycetota and Verrucomicrobiota were also highly connected, potentially supporting symbiotic relationships and ecosystem balance. Notably, Cyanobacteria, Sumerlaeota, and Thermoplasmatota exhibited negative correlations, mainly with fungal phyla such as Ascomycota, which could indicate competition for resources such as nutrients or space.

## 3. Discussion

This study evaluated the putative impact of organic and conventional farming systems on the prokaryotic and fungal diversity of soils associated with common bean crops and elucidated the implications of these practices on soil biodiversity. Several studies have investigated soil microbiomes by comparing conventional and organic farming systems, but they primarily focused on samples from a single year and did not always consider the impact of specific plant genotypes or cultivars [29,30,31]. Plant genotypes play a crucial role in shaping the root morphology and root exudate composition. Different genotypes can exhibit distinct root phenotypes—such as variations in length, branching patterns, and root hair density—as well as differences in the quantity and composition of the compounds released into the rhizosphere. These genotype-driven traits directly influence the surrounding microbial communities by selectively recruiting specific microorganisms and altering microbial activity, thereby affecting fundamental processes, such as nutrient acquisition [32]. As a result, plant genotype is a key factor in determining the structure and function of soil microbiomes, although it has not always been considered in microbiome-related studies. In this study, we analyzed soil microbial populations associated with the common bean genotype A25 over two consecutive years in crops in Northern Spain. By considering a specific bean variety and focusing on stable OTUs, we minimized sources of variation and ensured robust results.

The results showed that the type of farming system influences prokaryotic and fungal communities in distinct ways. For prokaryotes, richness was higher in CS, whereas fungal richness was higher in OS. The fungal community in OS exhibited higher evenness than that in CS, indicating a more balanced distribution of species. While some studies suggest that organic farming tends to increase the taxonomic richness, diversity, and heterogeneity of both prokaryotic and fungal soil communities compared to conventional farming [29,33,34], this finding is not always consistent. There are studies in which the type of farming system did not show a significant impact on bacterial communities [31], which seems to be more dependent on other factors, such as crop type, soil environmental conditions, or specific agricultural practices. These discrepant results highlight the complexity of sources of variation that affect soil microbial communities and underline the need to expand knowledge on this subject.

CS and OS differed in terms of bean crop intensity and inputs (fertilizers and pesticides) applied over the last six years. Both showed similar pH values (7.5) and texture (load) but differed in conductivity and for certain minerals, which can be attributed to the inorganic or chemical fertilization performed in CS (see Table S5, soil history). Fertilizers are important inputs for crops, particularly for conventional crop systems. The use of chemical fertilizers has been reported to change the abundance of microbial populations and stimulate their growth due to the nutrient supply added, but no significant influence on the richness and diversity of bacteria and fungi was reported [35]. Although we cannot attribute specific microbial differences to fertilization practices, they may nonetheless represent a potential source of variability between CS and OS microbiomes in our study.

In CS, two bean crops were annually developed, whereas OS supported one bean crop per year, involving rotation with a cereal crop. The benefits of crop rotation, which involves growing a sequence of different plant species on the same soil, are known to reduce soil erosion, improve soil structure and stability, disrupt the pest and pathogen lifecycle, and improve soil microbial biomass, community composition, and activity [36,37]. Thereby, studies have shown that the soil microbiome can improve responses to diseases through the production of antimicrobial compounds, microbial–microbial competition, or the activation of plant defence [38]. Common bean crops can be affected by soil-borne pathogens (e.g., *Aphanomyces*, *Fusarium*, *Pythium*, *Rhizoctonia*, and *Thielaviopsis* genera), causing damage to their roots, potentially leading to stunted growth, yellowing of leaves, and reduced yields [39]. In this regard, a higher incidence of plants showing symptoms consistent with root diseases was visually observed in CS suggesting a potential link between conventional farming and increased root health issues. Nevertheless, this observation was based solely on field-level visual assessment and was not supported by a detailed pathological analysis so further investigation would be required to confirm the presence and identity of any root pathogens involved. The control of bean root diseases through genetic resistance is complicated because several fungi can be involved and resistant genotypes are not available in all cases. Agronomic practices, including soil farming, to preserve biodiversity can help minimize the impact of root diseases.

CS and OS also differed in the application of synthetic fungicides and insecticides (see Table S5). These differences could be the main factor explaining the differences in biodiversity between the two soils. Azoxystrobin and Thiophanate-methyl are two active materials that were repeatedly used in CS as broad-spectrum fungicides and were used very intensively to control the fungal disease powdery mildew. Azoxystrobin inhibits mitochondrial respiration, whereas thiophanate-methyl inhibits mitosis and cell division. The main degradation product of thiophanate-methyl in the soil is carbendazim, which also has fungicidal properties. It has been described that both fungicides affect the soil microbiome [40,41,42]. Deltamethrin (a synthetic pyrethroid insecticide) and abamectin were two insecticides used in CS that negatively affect soil biodiversity, particularly in arthropods and microorganisms [43,44].

Within these communities, we observed contrasting trends in specific microbial genera. Notably, OS showed higher abundances of beneficial fungi crucial for plant health, including the genus *Trichoderma*, known for their use in biological control [45], and mycorrhizae, such as *Claroideglomus*, *Funneliformis*, *Rhizophagus*, *Diversispora*, and *Dominikia*, which may contribute to ecosystem resilience. In contrast, potentially phytopathogenic fungi, such as *Fusarium* and *Penicillium*, were more prevalent in CS, and mycorrhizae were barely undetectable. This is consistent with other studies in which arbuscular mycorrhizae were severely depressed under conventional farming [46] and aligns with the fact that bacterial communities in organic farming modulate fungal plant pathogens [47]. These differences suggest that organic farming practices in bean crops foster environments that are conducive to beneficial microbial communities.

Fungal lifestyles are aligned more closely with sustainability and environmental friendliness in organic farming (endophytes and animal pathogens; see Figure 3). In addition, processes such as sulphur and iron respiration, chlorate reduction, and fermentation, which are crucial for plant health, are more prevalent in OS. These functional differences are also reflected in other studies showing that OS can enhance microbial metabolic activities related to nutrient cycling and plant health [48]. In contrast, CS had higher levels of intracellular parasites, including phytopathogenic bacteria, indicating potential risks to plant health.

Regarding the interactions between phyla, our data suggest a direct influence of farming practices on microbial community dynamics. The networks established in CS exhibited striking patterns of inhibition and promotion, reflecting a complex interplay of interactions (both positive and negative). These findings suggest a community shaped by intense competition for resources and adaptation to external inputs such as agrochemicals [49,50]. In contrast, the predominance of positive interactions among fungi and bacteria in OS, along with a more balanced and diverse community characterized by the absence of dominant phyla, indicates a more cooperative and potentially resilient soil ecosystem [21]. This is consistent with the work of Lori et al. [51], who found that organic farming practices foster cooperative microbial communities, potentially facilitating nutrient cycling and plant growth [34]. The fungal phyla Rozellomycota and Ascomycota, along with the bacterial phylum Proteobacteria, played important roles in CS and OS networks, acting as keystone phyla. Their capacity to shape the networks, whether through mutualism or competitive exclusion, underscores their ecological importance. This should be validated in other common bean genotypes and environments.

Finally, this research increases understanding on soil microbial communities, highlighting the intricate relationships between farming practices, microbial diversity, and network interactions in a temperate oceanic climatic region. The idea that the soil microbiome is closely linked to soil and plant health is gaining traction in the scientific community, with several studies proposing the use of microbial community composition and diversity as biomarkers for assessing soil quality and sustainability of agricultural practices [52,53]. Expanding this research to other geographical regions, crops, or other common bean genotypes could reveal the specific microbial communities used as markers of healthy soil and crop system.

## 4. Materials and Methods

### 4.1. Experimental Design

Two loam soils located in Villaviciosa (Asturias, Spain; 43°28’27.0″ N, 5°26’30.1″ W) were analyzed in this study. This area has a temperate oceanic climate, characterized by mild temperatures, high humidity, and abundant rainfall throughout the year. The average annual temperature is approximately 13 °C, with moderate seasonal variations. One soil (CS) was managed following conventional farming systems in a tunnel greenhouse for the last 20 years. CS supported 11 bean crops (61% of soil occupation in terms of time) and 72 phytosanitary treatments to control pests and diseases during between 2018 and 2023 (Table S5). The use of fungicides to control powdery mildew as azoxystrobin (15 applications), thiophanate-methyl (7), and penconazole (3) is remarkable. Another soil (OS) was managed in the organic farming system for the last 6 years (2018–2023) in open field. Six bean crops corresponding to 50% of the OS occupation were developed during the 2018–2023 period. A total of 47 organic phytosanitary treatments were applied to bean crops to control pests and diseases (Table S5). Fertilization of the OS was performed using green manure with a common bean–ryegrass rotation system (bean in summer and ryegrass in winter, the ryegrass being incorporated into the soil as green manure). Both systems were mulched with plastic to control weeds.

### 4.2. Soil Sampling for Metabarcoding Analysis

Soil samples were taken close to the roots of bean line A25 with the help of a 5 cm diameter sampling probe. Line A25 is derived from an old cultivar obtained through local landrace selection [54]. This genotype has an indeterminate climbing growth habit and is classified as a market class fabada. Five replicates per soil (CS1, CS2, CS3, CS4, CS5, and OS1, OS2, OS3, OS4, and OS5) and year (2022 and 2023) were sampled in cross-transects at 30 cm depth and independently analyzed. Each replicate consisted of three independent sub-samples per plant. Samples were collected during the flowering stage, and irrigation was avoided during the previous days. The samples were frozen immediately after collection.

### 4.3. Soil Physicochemical Analyses and Soil Environmental Data

Physicochemical analyses of the CS and OS were conducted by the company KUDAM (Laboratorio Kudam S.L., Alicante, Spain) in 2022 and 2023. Each soil sample was harvested using a shovel at 40–50 cm depth as a mix of 8–10 subsamples spread across the field to capture variability. These analyses included the main physical parameters (texture, humidity, conductivity, and pH) and chemical measurements of chloride, sulphate, sodium, nitrogen, nitrate, phosphorus, potassium, calcium, manganese, and zinc. Each year, the entire bean crop cycle was monitored using soil sensors that recorded temperature and relative soil moisture every 15 min. From these data, the average soil temperature, average relative humidity, number of days with extreme soil temperatures (above 25 °C), and number of days with extreme drought conditions (relative humidity below 15%) were calculated (Figure S1).

### 4.4. Soil DNA Extraction and Sequencing

DNA was extracted from 1 g of each soil sample using a NucleoSpin Soil DNA Extraction Kit (Macherey-Nagel, Düren, Germany). The composition and structure of the microbial communities were assessed through amplification and sequencing of the V3-V4 variable regions of the 16S rRNA gene for prokaryotes and the internal transcribed spacer 2 (ITS2) region for fungi. The primers used for amplifying 16S rRNA were 341F (5′-CCTACGGGNGGCWGCAG-3′) and 785R (5′-GACTACHVGGGTATCTAATCC-3′), and the primers used to amplify ITS were ITS3 (5′-GCATCGATGAAGAACGCAGC-3′) and ITS4 (5′-TCCTCCGCTTATTGATATGC-3′). Amplification was performed after 25 cycles of PCR. Negative controls were included in each case to detect environmentally derived contaminants, and a Mock Community (Zymo Research, California, USA) was included as a positive control. The libraries were sequenced using an Illumina MiSeq (300×2).

### 4.5. Bioinformatics Processing

Raw demultiplexed forward and reverse reads were processed using the QIIME2 v2024.5 software [55]. The Dada2 v1.16 software [56] was used for read trimming, quality filtering (Phred quality value > 20), denoising, pair-end merging, and phylotype calling. Unique sequences were grouped into OTUs. Phylogenetic assessments were performed using Mafft v7 [57] and FastTree v2.1 [58]. Taxonomic assignment was performed using a Bayesian Classifier [59] and Silva database version 138 [60] for prokaryotes (99% OTUs full-length sequences, accessed in August 2022 and November 2023) and Unite database version 8 [61] for fungi (99% OTUs full-length sequences, accessed in August 2022 and November 2023). Species consistently observed in at least one replication per year were considered the core population of prokaryotes and fungi, respectively, associated with the common bean crop. Species that were exclusively associated with farming systems were also identified.

Sequences were deposited and accessible in the European Nucleotide Archive (ENA) at European Molecular Biology Laboratory—European Bioinformatics Institute (EMBL-EBI; under the accession number PRJEB80709.

### 4.6. Diversity and Statistical Analysis

To ensure comparability between samples and account for variability in sequencing depth, the data were normalized using the rarefaction method. Rarefaction plots were generated to assess how the diversity metrics changed when more sequences were included.

The alpha diversity metrics of richness, evenness, and Shannon index were estimated per year from the average values of the CS and OS replicates, respectively. Richness refers to the total number of OTUs observed. Evenness refers to the abundance of different species within a community and is estimated using Pielou’s evenness index [62]. Significant differences due to farming system (conventional vs. organic) and year (2022–2023) were estimated using the negative binomial model and the “MASS” R package [63] for richness, and the beta regression model in the “betareg” R package [64] for evenness. Pielou’s evenness indexes were compared between CS and OS samples using a Two-way ANOVA (for 16S) and Kruskal–Wallis (for ITS) with “stats” and “car” R packages [65]. The Shannon diversity index was computed using the “diversity” function from the “vegan” package in R [66]. For each sample, the function calculated the index using the formula H′ = −∑ (pi × ln(pi)), where pi is the proportion of individuals belonging to species i. In our study, OTUs were used as a proxy for species, and their relative abundances were derived from the sequencing read counts. The transposed abundance matrix was used as the input, ensuring that each column represented a sample and each row represented an OTU. This approach allowed us to quantify the diversity of both fungal and prokaryotic communities, considering both the richness and evenness of each sample.

Phylogenetic distances between OTUs were used to calculate beta diversity based on Jaccard similarity coefficient. This ecological metric informs about the microbial community’s structure. Permutational Multivariate Analysis of Variance (PERMANOVA) was used to estimate significant differences in beta diversity due to farming system and year using the “vegan” R package [66]. Principal coordinate analysis (PCoA) of the Jaccard distance matrix was performed to investigate the clustered samples. Finally, the Wilcoxon–Mann–Whitney test implemented in the “stats” R package was used to investigate putative significant differences between medians.

Correlation analyses and ecological networks were performed for common phyla detected in at least three of the total observations, irrespective of the farming system or year. To visualize the ecological networks, nodes and connections were implemented to represent the interactions between the graph components. Only correlations higher than 0.5 were accepted. The R packages used to generate the correlation matrices and network plots were “corr” [67], “corrplot” [68], “igraph” [69], and “PerformanceAnalytics” [70].

Statistical analyses were performed using the R software version 4.4.1 [71]. In all cases, assumptions of normality (Shapiro–Wilk test, “stats” R package) and homoscedasticity (Levene’s homogeneity of variance test, “car” R package) were studied before performing the corresponding statistical analyses. The significance threshold was set at *p* < 0.05.

### 4.7. Functional Analyses

Functional predictions of the prokaryotic community were performed using the FAPROTAX database [72] (accessed in November 2024) using the file of OTUs observed by the species as the input. Only species exclusive to each farming system were considered for the functional analyses.

Fungal OTUs were taxonomically parsed by ecological guild using the FUNGuild database [73] (accessed in November 2024) and its community-annotated database, processing the list of OTUs observed by species as input. According to the criteria proposed by Camacho-Sanchez et al. (2023) [74], only guilds defined as “probable” and “highly probable” were considered trustable for discussion. In addition, the list of functions assigned to each species was revised and a literature review was conducted to adjust the fungal classification into four categories: “Endophyte”, “Plant Pathogen”, “Animal or Fungal Parasite”, and “Saprotroph”. As for prokaryotes, only fungal species exclusive to each farming method were considered for functional analyses.

## 5. Conclusions

This study revealed the impact of organic and conventional farming systems on soil microbiome composition and diversity associated with common bean crops in Northern Spain. A core population of microorganisms consistently associated with common bean crops over two consecutive years was identified. In this work, organic farming significantly enhanced microbial diversity and promoted healthier soils compared to conventional farming. OS exhibited improved nitrogen metabolism, stronger positive interactions between fungi and bacteria, and a higher abundance of beneficial microorganisms, such as biocontrol fungi and mycorrhizae. In contrast, CS showed a higher prevalence of potentially phytopathogenic fungi and more complex competitive microbial interactions. These results highlight the potential of organic farming to boost soil diversity and enhance microbial network interactions, though further research is needed in different climatic regions and crop systems.

## Figures and Tables

**Table 1:** Prokaryotic (16S) and fungal (ITS) sequencing data summary per farming system and year.

Soil	Year	Seq.	Input	Input Passed Filter (%)	Denoised	Merged Input (%)	Non-Chimeric Input (%)	Observed OTUs
CS	2022	16S	120,179 ± 27,066	77.86	70,955 ± 16,364	34.04	33.64	634 ± 117
		ITS	160,773 ± 69,498	74.43	117,357 ± 45,972	68.57	67.09	167 ± 32
	2023	16S	91,369 ± 68,881	59.10	41,285 ± 32,190	27.62	27.08	453 ± 53
		ITS	48,594 ± 20,817	50.10	22,807 ± 12,277	41.15	40.51	147 ± 44
OS	2022	16S	86,807 ± 11,692	77.89	52,710 ± 7877	31.69	31.35	489 ± 78
		ITS	126,259 ± 34,298	74.43	93,871 ± 28,359	66.13	65.28	260 ± 76
	2023	16S	93,863 ± 55,541	54.53	39,946 ± 29,127	22.65	22.05	352 ± 7
		ITS	41,308 ± 10,520	54.66	20,731 ± 7278	43.98	43.44	217 ± 78

The data are presented as the average value of the conventional soil (CS) and the organic soil (OS) replicates and its standard deviation (SD). Input: number of reads before quality filtering. Input passed filter (%): percentage of reads retained after quality filtering and trimming. Denoised: number of reads retained after denoising steps, Merged input (%): percentage of merged reads (only merged reads are kept for further analysis). Non-chimeric input (%): percentage of reads retained after chimera removal.

**Table 2:** Statistical analysis of alpha and beta diversity for prokaryotic (16S) and fungal (ITS) communities in response to the farming system (conventional vs. organic) and year (2022–2023).

Diversity	Statistic Model	Organism	Coefficient	Z Value	F Value	Pr (>|z|)
Alpha diversity: Richness	Binomial negative	16S	Farming	2.78	-	5.50 × 10^−3^
			Year	3.38	-	7.31 × 10^−3^
			Farming:Year	0.06	-	0.95
		ITS	Farming	2.89	-	3.84 × 10^−3^
			Year	0.53	-	0.60
			Farming:Year	0.33	-	0.74
Alpha diversity: Evenness	Beta regresion	16S	Farming	1.54	-	0.12
			Year	0.46	-	0.64
			Farming:Year	2.47	-	0.01
		ITS	Farming	5.34	-	9.34 × 10^−8^
			Year	1.80	-	0.07
			Farming:Year	1.78	-	0.08
Alpha diversity: Shannon Index	ANOVA	16S	Farming	-	0.60	0.45
			Year	-	2.40	0.14
			Farming:Year	-	0.14	0.71
		ITS	Farming	-	27.31	8.33 × 10^−5^
			Year	-	0	0.99
			Farming:Year	-	2.10	0.17
Beta diversity	Permanova of Jaccard distances	16S	Farming	-	1.16	1.00 × 10^−3^
			Year	-	1.15	1.00 × 10^−3^
			Farming:Year	-	1.09	2.00 × 10^−3^
		ITS	Farming	-	3.67	1.00 × 10^−3^
			Year	-	2.20	1.00 × 10^−3^
			Farming:Year	-	1.96	3.00 × 10^−3^

The statistic model used in each case is indicated. ‘Farming’ represents the effect of farming system, ‘Year’ indicates the effect of annuity, and ‘Farming: Year’ represents the interaction between farming and sampling year. Significance *p* < 0.05.

**Table 3:** The abundance of prokaryotic taxa with significant differences between the conventional soil (CS) and organic soil (OS) over the two years.

	2022			2023		
	CS	OS		CS	OS	
Taxonomy	Mean ± SD	Mean ± SD	*p*-Value	Mean ± SD	Mean ± SD	*p*-Value
Uncultured verrucomicrobia (g)	2.49 ± 6.91	0	2.00 × 10^−3^	2.73 ± 8.79	0	1.24 × 10^−2^
Uncultured planctomycete (g)	4.33 ± 13.6	0.38 ± 2.24	2.59 × 10^−2^	2.00 ± 7.28	0	4.33 × 10^−2^
Uncultured chthoniobacter (g)	0	2.31 ± 7.52	1.13 × 10^−3^	0	1.92 ± 6.95	2.34 × 10^−2^
Uncultured bacteroidetes (g)	0.13 ± 1.12	4.06 ± 13.56	8.61 × 10^−3^	0.45 ± 2.54	3.61 ± 10.19	4.10 × 10^−2^
Uncultured UTBCD1 (g)	0.85 ± 2.68	0	4.28 × 10^−2^	2.91 ± 10.15	0	4.21 × 10^−2^
Uncultured adlerbacteria (g)	1.05 ± 3.50	0.14 ± 0.95	2.77 × 10^−2^	1.42 ± 4.65	0	6.87 × 10^−3^
Uncultured A4b (g)	1.23 ± 4.71	0.24 ± 1.31	2.00 × 10^−2^	0.89 ± 3.96	0.12 ± 0.99	3.13 × 10^−2^
Uncultured bacteriap25 (g)	1.43 ± 5.02	0.03 ± 0.26	7.79 × 10^−3^	1.40 ± 4.44	0	2.30 × 10^−2^
Candidatus uhrbacteria (g)	1.2 ± 3.88	0	2.28 × 10^−2^	0.69 ± 2.01	0	4.25 × 10^−2^
Uncultured desulfuromonas (g)	0	4.55 ± 13.57	2.25 × 10^−2^	0	2.84 ± 8.78	4.21 × 10^−2^
Uncultured vicinamibacteraceae (g)	0.08 ± 0.67	3.08 ± 8.76	4.57 × 10^−3^	0	3.11 ± 10.15	6.87 × 10^−3^
Uncultured planctomycete (g)	0.00 ± 0.00	1.48 ± 5.77	7.17 × 10^−3^	0.44 ± 2.56	2.99 ± 8.70	4.29 × 10^−2^
Ferruginibacter (g)	0.00 ± 0.00	2.49 ± 7.39	3.72 × 10^−3^	0.00 ± 0.00	3.40 ± 10.82	2.31 × 10^−2^
Rokubacteriales (g)	3.13 ± 11.44	1.75 ± 9.98	3.87 × 10^−2^	3.30 ± 12.57	1.43 ± 9.32	6.48 × 10^−3^
SJA-28 (g)	0	2.24 ± 6.46	2.28 × 10^−2^	0	2.94 ± 7.59	2.22 × 10^−2^
Uncultured simkaniaceae (g)	1.65 ± 5.92	0.07 ± 0.54	4.87 × 10^−2^	1.18 ± 4.49	0	2.27 × 10^−2^
Uncultured sutterellaceae (f)	0	6.23 ± 15.06	4.95 × 10^−5^	0	4.63 ± 16.63	2.34 × 10^−2^
Roseiflexaceae (f)	2.34 ± 5.79	0.10 ± 1.16	1.46 × 10^−6^	0.84 ± 3.46	0	7.31 × 10^−3^
Gemmatimonadaceae (f)	2.96 ± 9.12	0	3.39 × 10^−4^	2.83 ± 8.88	0	6.87 × 10^−3^

The mean abundance (number of observed OTUs) from biological replicates and standard deviation (SD) are shown. Taxa were identified to the maximum identification level: s, species; g, genus; o, order; f, family. *p*-values indicate the statistical significance of the differences between treatments for each year (Mann–Whitney U test).

**Table 4:** The abundance of fungal taxa with significant differences between conventional soil (CS) and organic soil (OS) over the two years.

	2022			2023					
	CS	OS		CS	OS				
Taxonomy	Mean ± SD	Mean ± SD	*p*-Value	Mean ± SD	Mean ± SD	*p*-Value	Guild	Confidence Ranking	Source
*Botryotrichum spirotrichum* (s)	55.00 ± 34.66	0	7.49 × 10^−3^	149.60 ± 95.87	2.00 ± 4.47	9.70 × 10^−3^	Saprotroph	P	[11,23]
*Claroideoglomus claroideum* (s)	0.28 ± 1.44	7.78 ± 21.93	4.11 × 10^−5^	0.03 ± 0.39	2.32 ± 8.35	5.53 × 10^−5^	Arbuscular Mycorrhizal	HP	[11,23]
*Emmonsiellopsis coralliformis* (s)	2.20 ± 1.79	0	2.48 × 10^−2^	38.20 ± 21.78	0	7.49 × 10^−3^	Saprotroph	P	[23]
*Exophiala equina* (s)	0	4.90 ± 9.15	1.49 × 10^−2^	0	10.00 ± 14.66	1.49 × 10^−2^	Animal Parasite	P	[11,23,24]
*Funneliformis mosseae* (s)	0.13 ± 0.91	3.23 ± 10.92	3.30 × 10^−7^	0.03 ± 0.32	1.80 ± 10.46	1.77 × 10^−2^	Arbuscular Mycorrhizal	HP	[23,25]
*Pyrenochaetopsis leptospora* (s)	0.04 ± 0.20	5.60 ± 15.62	1.77 × 10^−2^	0	7.52 ± 22.13	2.49 × 10^−3^	Endophyte	Ps	[11,23,26]
*Rhizophagus fasciculatus* (s)	0	5.90 ± 21.33	4.28 × 10^−2^	0.40 ± 1.81	7.48 ± 18.33	4.09 × 10^−2^	Arbuscular Mycorrhizal	HP	[23,25]
Acrocalymma (g)	127.60 ± 119.94	7.80 ± 11.37	1.12 × 10^−2^	41.80 ± 24.72	154.60 ± 75.45	2.16 × 10^−2^	-	-	-
Ciliophora (g)	0.31 ± 1.62	0.69 ± 2.60	1.73 × 10^−2^	0.81 ± 3.11	0.28 ± 2.34	2.79 × 10^−3^	-	-	-
Cylindrocarpon (g)	0.25 ± 1.12	5.25 ± 5.75	9.97 × 10^−5^	0.05 ± 0.22	4.80 ± 7.90	3.20 × 10^−2^	Plant Pathogen	P	[11,23]
Diversispora (g)	0	1.83 ± 5.33	3.65 × 10^−3^	0	0.92 ± 3.39	2.34 × 10^−2^	Arbuscular Mycorrhizal	HP	[23,25]
Dominikia (g)	0	2.88 ± 8.70	2.09 × 10^−2^	0	1.72 ± 4.06	4.12 × 10^−2^	Arbuscular Mycorrhizal	HP	[25]
Pyrenochaeta (g)	1.10 ± 3.82	4.85 ± 7.38	3.01 × 10^−2^	0.30 ± 1.34	7.25 ± 12.61	1.64 × 10^−2^	Saprotroph	HP	[11,23]
Ramicandelaber (g)	26.48 ± 69.44	0.60 ± 1.68	3.37 × 10^−2^	22.44 ± 46.03	0.16 ± 0.80	3.66 × 10^−2^	Saprotroph	P	[11,23]
Rhizophagus (g)	0.07 ± 0.54	2.76 ± 9.07	3.97 × 10^−4^	0.00	0.69 ± 2.96	1.32 × 10^−2^	Arbuscular Mycorrhizal	HP	[25]
Talaromyces (g)	5.78 ± 14.64	2.80 ± 15.89	8.30 × 10^−3^	16.67 ± 52.81	3.09 ± 11.29	7.16 × 10^−3^	Saprotroph	Ps	[27]
Tetracladium (g)	0	4.05 ± 7.25	4.53 × 10^−3^	0	4.65 ± 9.48	1.98 × 10^−2^	Saprotroph	P	[11,23]
Agaricales (o)	0	2.73 ± 8.33	4.19 × 10^−2^	0	0.63 ± 2.04	4.19 × 10^−2^	Saprotroph	Ps	[28]
Helotiales (o)	0	1.13 ± 2.19	4.17 × 10^−4^	0	4.70 ± 13.79	6.17 × 10^−3^	-	-	-
Rhizophydiales (o)	0	8.77 ± 23.04	3.26 × 10^−9^	0.01 ± 0.09	0.42 ± 2.15	3.01 × 10^−2^	-	-	-
Didymellaceae (f)	0.08 ± 0.28	4.36 ± 8.64	4.56 × 10^−2^	0.32 ± 1.11	5.72 ± 8.66	4.94 × 10^−3^	-	-	-
Glomeraceae (f)	0	1.33 ± 4.60	1.91 × 10^−4^	0.14 ± 1.43	2.97 ± 7.89	8.04 × 10^−6^	Arbuscular Mycorrhizal	HP	[25]
Nectriaceae (f)	0.67 ± 1.91	9.67 ± 11.49	1.01 × 10^−2^	2.47 ± 5.46	38.73 ± 66.26	3.67 × 10^−2^	Saprotroph	Ps	[27]

The mean abundance (number of observed OTUs) from biological replicates and standard deviation (SD) are shown. Taxa are identified to the maximum identification level: s, species; g, genus; o, order; f, family. *p*-values indicate the statistical significance of the differences between treatments for each year (Mann–Whitney U test). Guild and functional confidence were assigned according to FUNGuild, with confidence rankings (HP, highly probable; P, probable; Ps, possible) indicating the probability of the assigned guild.

## Data Availability

The data of this study are open and available in the ENA at EMBL-EBI under the accession number PRJEB80709.

## Supplementary Materials

The following supporting information can be downloaded at: https://www.mdpi.com/article/10.3390/plants14091359/s1, Figure S1: Soil environmental data for conventional soil (CS) and organic soil (OS) in 2022 and 2023; Figure S2: Alpha rarefaction plots for 16S and ITS samples per farming system and year; Figure S3: Histogram distribution of prokaryotic functions predicted by FAPROTAX; Table S1: Physicochemical analyses of CS and OS in 2022 and 2023; Table S2: Abundance of 1141 prokaryotic taxa in conventional soil (CS) and organic soil (OS) per year; Table S3: Abundance of 622 fungal taxa in conventional soil (CS) and organic soil (OS) per year; Table S4: Spearman correlation matrix of microbial phyla; Table S5: Chronology of sowings and applications of chemical treatments in common bean crop soils under conventional and organic farming system.
