# Supplementary material for: Impact of Farming System on Soil Microbial Communities Associated with Common Bean in a Region of Northern Spain
Source: Plants (Basel). 2025 Apr 30;14(9):1359. doi: 10.3390/plants14091359 (PMC12073228; doi:10.3390/plants14091359)
Supplement: Supplementary file 1 [file plants-14-01359-s001.zip › Additional_File1_Supplementary_Figures_R1.pdf]

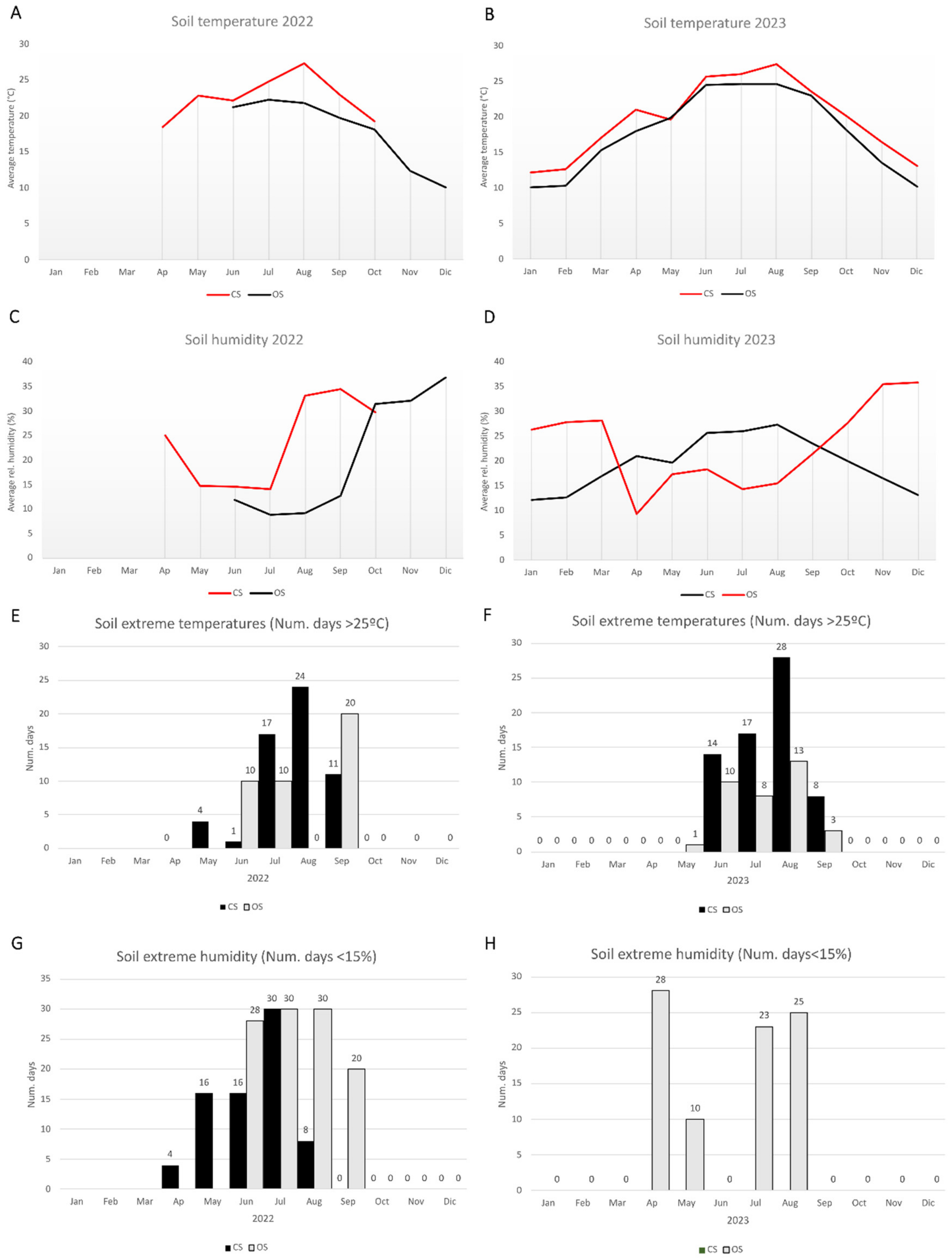

***Figure S1. Soil environmental data for conventional soil (CS) and organic soil (OS) in 2022 and 2023**. General records of soil temperature in 2022 (A) and 2023 (B) and soil humidity in 2022 (C) and 2023 (D). Number of days with extreme soil temperatures (above 25ºC) in 2022 (E) and 2023 (F), and number of days with drought (humidity below 15%) in 2022 (G) and 2023 (H) are also shown.*

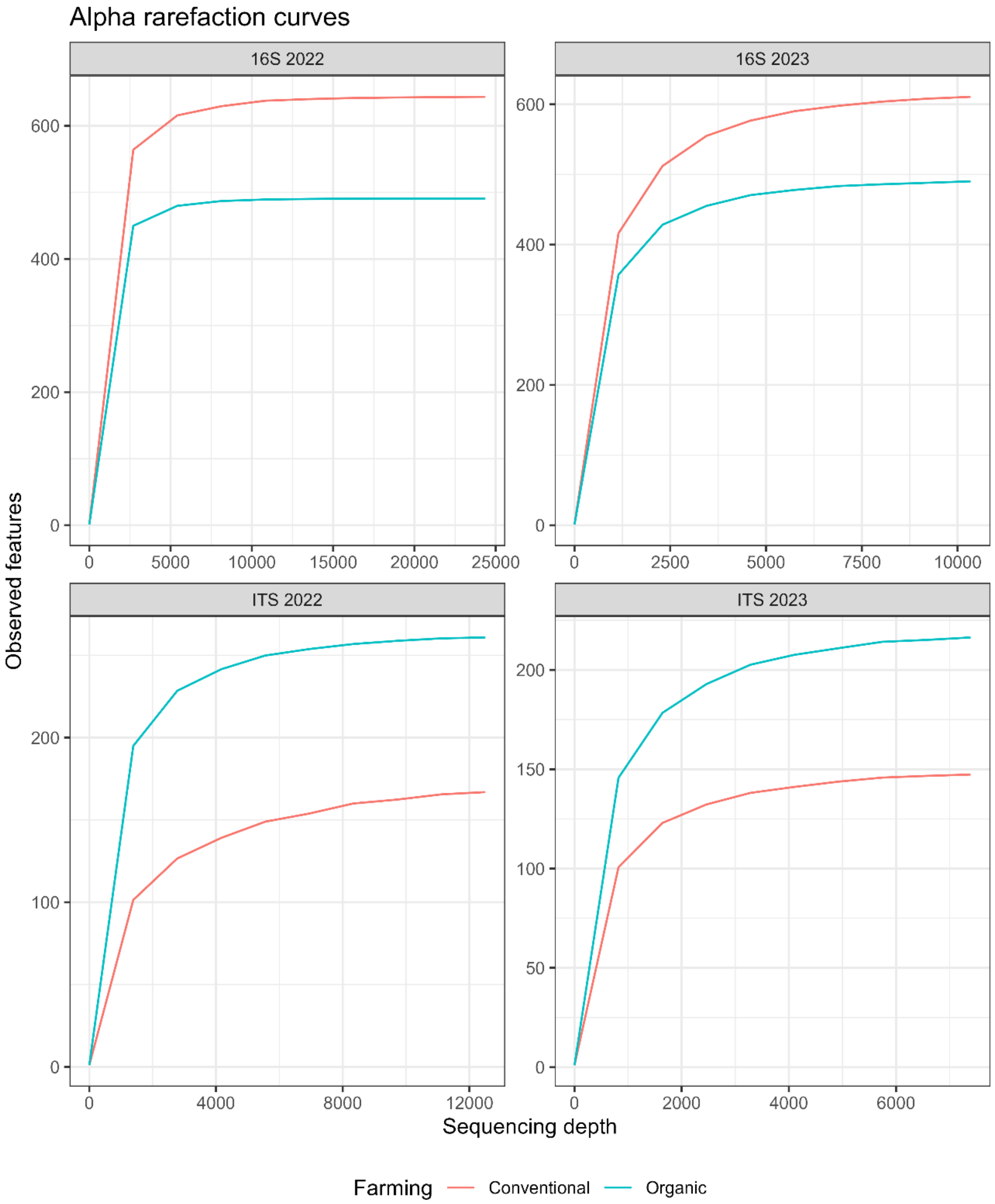

Figure S1. Alpha rarefaction plots for 16S and ITS samples per farming system and year*. X axis represents sequencing depth and Y axes de average observed features per treatment.* For 16S *the* sequencing depth *was* 24,360 reads in 2022 and 10,339 reads in 2023*; for* ITS the sequencing depth was 12,508 *reads* in 2022 and 7,390 in 2023

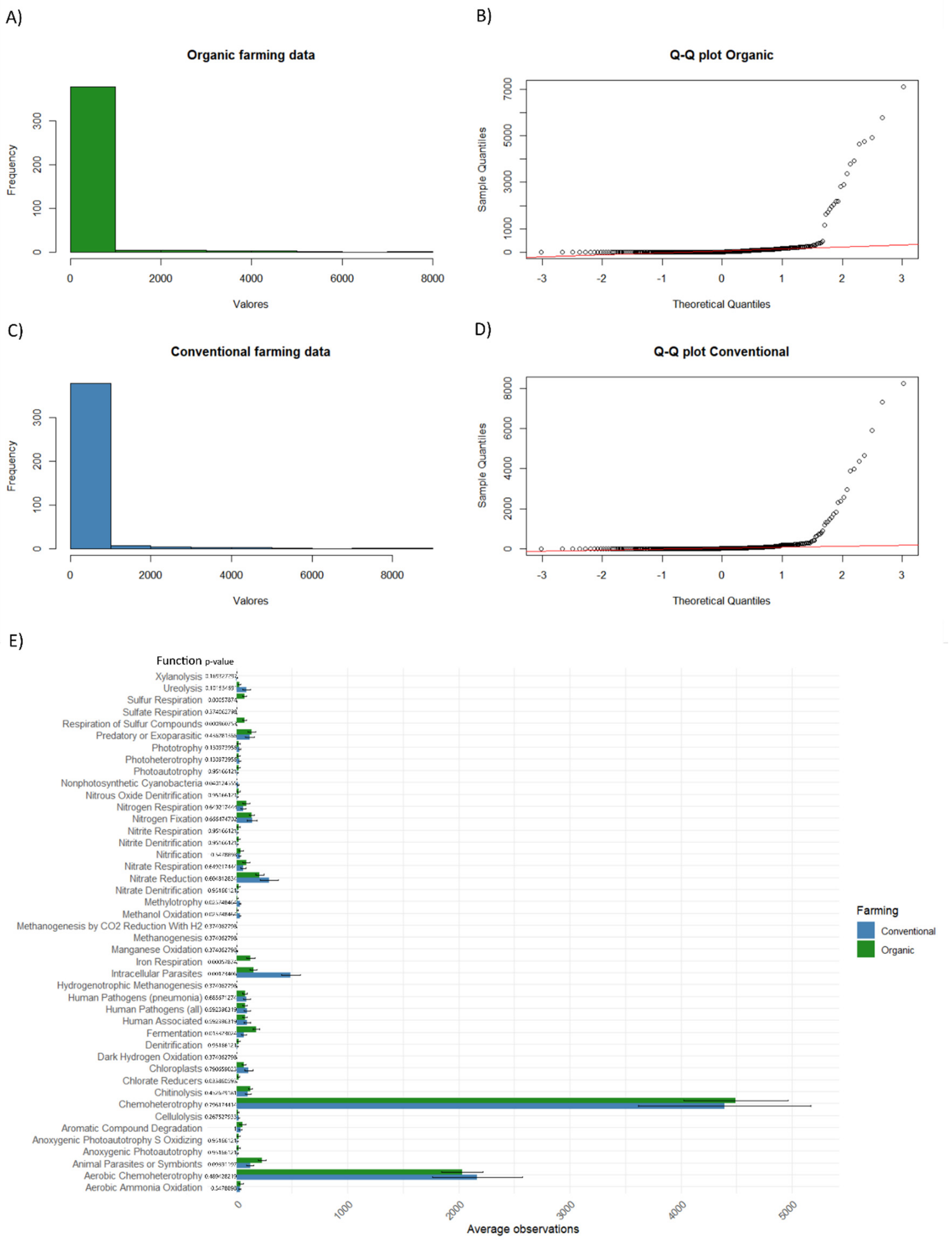

Figure S3. Histogram distribution of prokaryotic functions predicted by FAPROTAX*. A) For organic soil (OS) and B) its Q-Q plot. C) for conventional soil (CS) and D) its Q-Q plot. E) All functions predicted for prokaryotic species by FAPROTAX database in CS and OS. P values according to Mann Whitney U test are indicated at the right side of the function description. Bars represent average observations of replicates. Standard deviation is also indicated.*
